# Metabolic abnormalities, recurrence risk, patient and stone characteristics in calcium-based pediatric stone formers: is there any association?

**DOI:** 10.1007/s00345-026-06283-7

**Published:** 2026-03-19

**Authors:** Abdullah A. Sobh, Ahmed M. Shoma, Ahmed Abdelhalim, Abdelwahab Hashem, Amr A. Elsawy, Wael I. Mortada, Kareem A. Nabieh, Nasr A. El-Tabey

**Affiliations:** 1 Urology Department, Urology and Nephrology Center, Mansoura University, Mansoura, Egypt; 2 Department of Urology, West Virginia University, Morgantown, WV 26506 USA; 3 Faculty of Medicine, Delta University for Science and Technology, Dakahlia, Egypt; 4 Clinical Chemistry Laboratory, Urology and Nephrology Center, Mansoura University, Mansoura, Egypt

**Keywords:** Pediatric urolithiasis, Stone recurrence, Metabolic abnormalities, 24-hour urine collection

## Abstract

**Purpose:**

To assess the association between serum and urinary metabolic abnormalities, stone recurrence risk, and patient and stone characteristics in calcium-based pediatric stone formers; aiming to refine the indication for metabolic evaluation in pediatric urolithiasis population.

**Patients and methods:**

The study included 80 pediatric patients < 18 years with a history of intervention for calcium-based stones at the Urology and Nephrology Center, Mansoura University, Egypt, between 2001 and 2022. Electronic database was reviewed for patient and stone characteristics. Serum and 24-hour urinary metabolic evaluation was done. The primary outcome was to assess the association between serum and urinary metabolic abnormalities, and patient and stone characteristics. A secondary outcome was to assess risk factors associated with stone recurrence.

**Results:**

Serum and urinary metabolic abnormalities were present in 31.25% and 73.75% of the study population, respectively. There was no significant difference between normal and abnormal groups of serum and 24-hour urine chemistry in relation to all assessed patient and stone characteristics. However, there was a significant association between stone laterality and risk of recurrence (HR: 3.801, 95% CI: 1.239–11.662, ***p*** = 0.02); being ~ 3.8 times higher with bilateral urolithiasis (78.3% vs. 21.8% with unilateral urolithiasis).

**Conclusion:**

Routine serum and 24-hour urinary metabolic evaluation is still indicated in all pediatric calcium-based stone formers; being a high-risk population. However, the recurrence risk is higher in patients with bilateral urolithiasis. So, in case of limited resources, metabolic evaluation could be restricted to this group of patients only.

## Introduction

Pediatric stone disease is a significant clinical issue in various parts of the world, like the Middle East, South Asia, and North Africa. Although it was assumed to be rare in industrialized countries, epidemiology studies have shown that the incidence of pediatric urolithiasis is also increasing in the western world, especially in girls, children of white race, and older children [1]. According to literature, the prevalence of pediatric urolithiasis ranges from 1% to 5% in industrialized countries and from 5% to 15% in developing countries. Socioeconomic factors, dietary habits, ethnic, genetic, and geographic variations all contribute to this discrepancy [2].

Recent stone analyses in children show a similar distribution to adults where approximately 75% to 80% of stones are composed of predominantly calcium oxalate, 5% are predominantly calcium phosphate, 10% to 20% are struvite, and 5% are pure uric acid [3].

Urolithiasis in the pediatric population is associated with a higher rate of metabolic abnormalities and urinary tract anomalies, with more than 50% having metabolic abnormalities, and about 30% having urinary tract anomalies, most commonly ureteropelvic junction obstruction. Therefore, it’s critical to identify the risk factors that contribute to the etiology and to take appropriate measures to prevent stone recurrence [4]. Medical prophylaxis, according to the underlying etiology, has been shown to reduce the frequency of stone disease recurrence [5].

Previous studies reported a higher prevalence of serum and urine biochemical abnormalities among pediatric urinary stone formers. Underlying metabolic abnormalities are thought to contribute to the high recurrence risk observed in pediatric urolithiasis. Assuming that residual stone burden and medical prophylaxis influence the recurrence rate in pediatric urolithiasis and that it has close relationship with metabolic abnormalities, recurrence rates may be decreased with successful surgery and adequate prophylaxis [6]. Metabolic evaluation is highly recommended following stone removal in order to identify and treat any underlying metabolic abnormality [7]. According to the European Association of Urology (EAU) guidelines, it is recommended to collect the stone material for analysis to classify the stone type, then to complete a metabolic evaluation based on stone analysis, in all children [8]. American Urological Association (AUA) guidelines also recommend a complete urinary metabolic evaluation in all pediatric stone patients after the first stone event due to a high incidence of metabolic abnormalities [9].

However, 24-hour urinary metabolic evaluation has several limitations. First, collection of 24-hour urine for metabolic evaluation is not straightforward, particularly among non-toilet-trained children and often requires urethral catheterization., Second, 24-hour metabolic evaluation has limited availability and represents a substantial financial burden, particularly in developing countries where the stone disease is more prevalent and resources are limited [10].

To overcome some of these limitations, we hypothesize that the indications for 24-hour urinary metabolic evaluation could be restricted to children at highest risk for stone recurrence to increase its yield and limit the associated cost. So, in this study, we assume that there may be an association between serum and urinary metabolic abnormalities and certain patient and stone characteristics. Furthermore, recurrence risk will be assessed in relation to these patient and stone characteristics, and to serum and urinary metabolic abnormalities.

## Patients and methods

This is a non-concurrent cohort study that included pediatric stone formers < 18 years with history of intervention for stone disease at the Urology and Nephrology Center, Mansoura University, Egypt, from 2001 to 2022. Patients were excluded if they didn’t have stone analysis, or if they proved to be non-calcium-based stone formers based on available stone analysis. Patients who missed follow-up and didn’t complete evaluation, and those with recurrent stone disease needing surgical intervention at time of evaluation were also excluded.

**Fig. 1:**
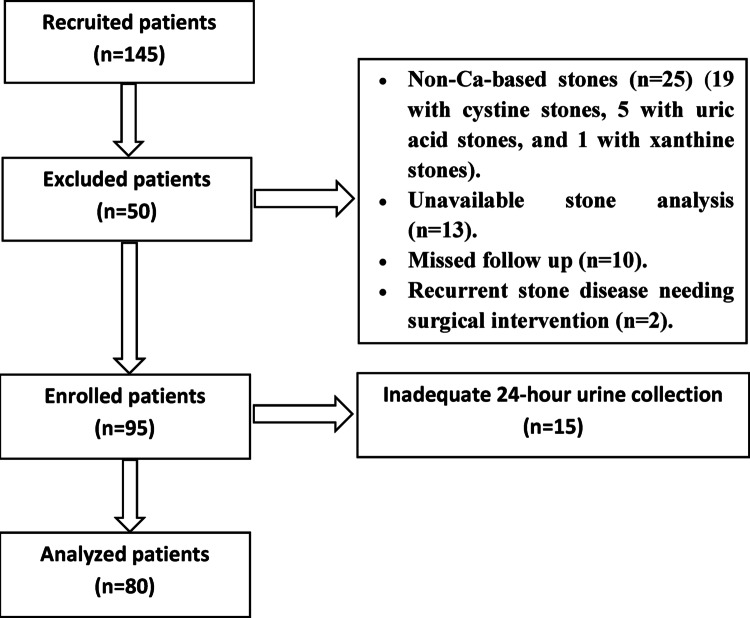
Flow chart of the study cases

Patients were recruited to participate in the study from May 2022 to January 2023. Contact data of 482 patients were retrieved from Patient Information System. Of them, 334 patients could be reached. Among the contacted patients, 145 presented for an outpatient clinic visit. Fifty patients were excluded. The remaining 95 patients were then eligible for further metabolic evaluation. After 24-hour urinary metabolic evaluation, 15 patients had inadequate urine collection based on 24-hour urinary creatinine, and hence were also excluded. Therefore, only 80 patients were subjected to further analysis (Fig. 1).

Eligible patients were subjected to comprehensive metabolic evaluation after stone-free state was confirmed by radiological evaluation (ultrasound, KUB +/- NCCT). Metabolic evaluation was performed at least twenty days from the last stone episode in order not to be biased by atypical diet, fluid intake, activity, and renal function [8]. Random urine samples were checked first for urine crystals, pus cells, and pH. Urine pH is affected by metabolic abnormalities as well as urinary infection [11]. So, UTI was treated first if present.

Serum metabolic evaluation included creatinine, calcium, phosphorus, uric acid, albumin, pH, and bicarbonate. Serum calcium was corrected in case of hypoalbuminemia according to the formula: Corrected Serum Calcium (mg/dl) = (0.8 * (Normal Albumin - Patient’s Albumin)) + Serum Calcium. Normal albumin was considered 4 gm/dl [12, 13]. Serum chemistry was considered abnormal if one or more of the measured parameters were abnormal according to the pediatric reference ranges.

Twenty-four-hour urine collection was performed once for eligible patients while on regular diet [14], and was examined for creatinine, volume, pH, calcium, citrate, oxalate & uric acid. Reference values were used according to pediatric age group [15–17]. Twenty-four-hour urine chemistry was considered abnormal if one or more of the measured parameters were abnormal.

Electronic database was reviewed for clinical, laboratory and radiological data of the included 80 patients; including age of 1 st presentation with stone disease, BMI, family history, stone characteristics on 1 st episode, presence or absence of urinary tract anomalies, stone recurrence and number of stone recurrence episodes. The stone characteristics which were evaluated included stone number, volume, density, laterality, and complexity. Stone volume (in cm^3^) was measured directly from CT software. Stone density was measured in Hounsfield units (HU) on NCCT. Obstruction was presented as grade of hydronephrosis (HN) according to Society of Fetal Urology (SFU) grading system. Stone complexity was assessed according to STONE nephrolithometry and URS scores [18, 19].

The primary outcome was to assess the association between serum and urinary metabolic abnormalities in calcium-based pediatric stone formers, and patient and stone characteristics. A secondary outcome was to assess risk factors associated with stone recurrence in this population. Such association will help limit performing metabolic evaluation to patients with high prevalence of metabolic abnormalities and high risk for recurrence.

### Statistical analysis and data interpretation

Data were coded, processed and analyzed using the Statistical Package for Social Sciences (SPSS) program version 25. Qualitative data were presented by frequency tables (number and percentage). Numerical data were presented by means ± standard deviation (SD) if normally distributed and by median and interquartile range (IQR) if non-normally distributed. Chi square or Fisher’s exact test was used to test the significance of categorical data as appropriate. Independent samples T test and Mann-Whitney test were used to test the significance of continuous and ordinal data as appropriate. The results were considered significant when the probability of error is less than 5% (*p* < 0.05). Significant predictors in univariate analysis were integrated into cox regression model and hazard ratios (HR) and their 95% confidence intervals (CI) were calculated. The results were considered significant when the probability of error is less than 5% (*p* < 0.05).

### Ethical considerations

Data were used only for the purpose of this research study after informing patients and their caregivers about it. Study protocol was approved by Institutional Research Board (IRB) of Mansoura Faculty of Medicine, Mansoura university on 25/9/2021 (Code Number: MS.21.09.1657).

## Results

### Baseline patient and stone characteristics

Patients’ demographics and stone and kidney characteristics are summarized in Table 1. The age of patients at time of recruitment and evaluation ranged from 2 to 18 years, with a mean of 9.96 years. The age of 1 st stone episode ranged from 1 to 17 years, with a median of 6 years. Age groups according to age of 1 st stone episode were as follows: toddlers from 1 to 2 years, preschoolers from 3 to 5 years, school-aged children from 6 to 12 years, and teens from 13 to 17 years. Regarding BMI, patients were also divided into 2 groups: normal and obese, based on World Health Organization (WHO) BMI-for-age charts. The interval between diagnosis of the first stone episode and time of evaluation ranged from 1 month to 134 months with a median of 38 months. The interval between last stone episode and time of evaluation ranged from 1 month and 125 months with a median of 28.5 months.

Stones were considered pure calcium-based if calcium component was > 70%, and this was present in 54 patients (67.5%); 33 (41.25%) Ca-oxalate, 1 (1.25%) Ca-phosphate, 3 (3.75%) Ca-carbonate, and 17 patients (21.25%) with more than one calcium-based components. The other 26 patients (32.5%) had mixed stones (calcium component > 50% and < 70%).

Urinary tract anomalies were present in 14 patients (17.5%); 5 patients (6.25%) had UPJO (2 right, 1 left, and 2 bilateral), 3 patients (3.75%) had medullary sponge kidney and nephrocalcinosis, 2 patients (2.5%) had neuropathic bladder (one of them also had horseshoe kidney, and the other had bilateral vesicoureteric reflux), 2 patients (2.5%) had duplex system (1 right and 1 bilateral), 1 patient (1.25%) had unilateral renal agenesis, 1 patient (1.25%) had ectopic ureter, and 1 patient (1.25%) had history of epispadias-exstrophy complex.

**Table 1:** Patients’ demographics and stone and kidney characteristics

Demographic/Characteristic	Distribution
Age at evaluation (years): mean ± SD	9.96 ± 4.33
Gender: number (percentage)	
Male Female	52 (65%) 28 (35%)
BMI (Kg/m^2^): mean ± SD	18.57 ± 3.76
BMI group: number (percentage)	
Normal Obese	63 (78.75%) 17 (21.25%)
Recurrence: number (percentage)	
No Yes	50 (62.5%) 30 (37.5%)
Recurrence rate: number (percentage)	
None Once More than once	50 (62.5%) 22 (27.5%) 8 (10%)
Age at 1st episode (years): median (IQR)	6 (3.25–9.75)
Age group: number (percentage)	
Toddlers Preschoolers School-aged Teens	14 (17.5%) 20 (25%) 38 (47.5%) 8 (10%)
Family history: number (percentage)	
Negative Positive	37 (46.25%) 43 (53.75%)
Management: number (percentage)	
URS PCNL ESWL only More than one treatment modality Open Cystolitholapaxy	6 (7.5%) 44 (55%) 2 (2.5%) 18 (22.5%) 9 (11.25%) 1 (1.25%)
Stone composition: number (percentage)	
Pure Mixed	54 (67.5%) 26 (32.5%)
Stone volume (cm^3^): median (IQR)	1.27 (0.59–2.88)
Stone number: median (IQR)	3 (2–4)
Stone density (HU): mean ± SD	886.44 ± 384.91
Stone location: number (percentage)	
Ureteric Renal Combined Vesical	4 (5%) 55 (68.75%) 19 (23.75%) 2 (2.5%)
Laterality: number (percentage)	
Unilateral Bilateral Vesical	55 (68.75%) 23 (28.75%) 2 (2.5%)
Hydronephrosis: number (percentage)	
No Grade 1–2 Grade 3–4	25 (31.25%) 40 (50%) 15 (18.75%)
STONE score: median (IQR)	6 (6–7)
Urinary tract anomalies: number (percentage)	
Absent Present	66 (82.5%) 14 (17.5%)

### Primary outcome

#### Serum metabolic abnormalities

Among the included 80 patients, 3 patients (3.75%) had high serum creatinine, 3 patients (3.75%) had hypoalbuminemia, 7 patients (8.75%) had hypocalcemia, and no patients were hypercalcemic. Regarding serum phosphorus, 3 patients (3.75%) were hypophosphatemic and 4 patients (5%) had hyperphosphatemia. Hyperuricemia was present in 5 patients (6.75%). As for serum pH and bicarbonate, 10 patients (12.5%) had metabolic acidosis and 1 patient (1.25%) had metabolic alkalosis. Collectively, 55 (68.75%) had normal serum chemistry and 25 (31.25%) were abnormal.

#### 24-hour urinary metabolic abnormalities

Among the included 80 patients, 3 patients (3,75%) had low urine volume, 25 patients (31.25%) had lower-than-normal pH, and no patients had higher-than-normal pH. Hypercalciuria was present in 28 patients (35%). Ten patients (12.5%) had hyperuricosuria. Hyperoxaluria and hypocitraturia were present in 27 (33.75%) and 6 (7.5%) patients, respectively. Collectively, 21 patients (26.25%) had normal 24-hour urine chemistry and 59 patients (73.75%) were abnormal.

#### Combined serum and 24-hour urinary metabolic abnormalities

Considering serum and 24-hour urinary metabolic abnormalities together, only 19 patients (23.75%) had abnormal both serum and 24-hour urine, and the remaining 61 patients (76.25%) had normal either or both of them.

Then we had three groups of metabolic evaluation results to be analyzed; serum, 24-hour urine, and combined serum and 24-hour urine. Each of these groups was analyzed for possible significant association with assessed patient and stone characteristics. Results of this analysis are summarized in Tables 2, 3 and 4. There was no significant association between abnormal groups of serum, 24-hour urine or combined serum and 24-hour urine and any of the assessed patient and stone characteristics.

**Table 2:** Analysis of serum metabolic evaluation in relation to patient and stone characteristics

Parameter	Normal Serum Metabolic Evaluation (n = 55)	Abnormal Serum Metabolic Evaluation (n = 25)	*p* value
Gender: N (%)+			0.53
Male Female	37 (71.2%) 18 (64.3%)	15 (28.8%) 10 (35.7%)	
BMI (Kg/m^2^): mean ± SD*	18.17 ± 3.61	19.47 ± 3.98	0.15
BMI group: N (%)+			0.69
Normal Obese	44 (69.8%) 11 (64.7%)	19 (30.2%) 6 (35.3%)	
Age at 1st episode (years): median (IQR)#	6 (4–10)	6 (4-8.5)	0.65
Age group: N (%)**			0.85
Toddlers Preschoolers School-aged Teens	11 (78.6%) 14 (70%) 25 (65.8%) 5 (62.5%)	3 (21.4%) 6 (30%) 13 (34.2%) 3 (37.5%)	
Recurrence: N (%)+			0.42
No Yes	36 (72%) 19 (63.3%)	14 (28%) 11 (36.7%)	
Recurrence rate: N (%)**			0.55
None Once More than once	36 (72%) 13 (59.1%) 6 (75%)	14 (28%) 9 (40.9%) 2 (25%)	
Family history: N (%)+			
Negative Positive	26 (70.3%) 29 (67.4%)	11 (29.7%) 14 (32.6%)	
Urinary tract anomalies: N (%)+			0.69
Absent Present	46 (69.7%) 9 (64.3%)	20 (30.3%) 5 (35.7%)	
Stone volume (cm^3^): median (IQR)#	1.1 (0.54–2.96)	1.6 (0.77–2.6)	0.47
Stone number: median (IQR)#	3 (2–5)	2 (2-4.5)	0.74
Stone density (HU): mean ± SD*	876.82 ± 384.24	907.60 ± 393.47	0.74
Stone location: N (%)**			0.06
Ureteric Renal Combined Vesical	2 (50%) 35 (63.6%) 16 (84.2%) 2 (100%)	2 (50%) 20 (36.4%) 3 (15.8%) 0 (0%)	
Laterality: N (%)**			0.31
Unilateral Bilateral Vesical	35 (63.6%) 18 (78.3%) 2 (100%)	20 (36.4%) 5 (21.7%) 0 (0%)	
Hydronephrosis: N (%)**			0.85
No Grade 1–2 Grade 3–4	16 (64%) 28 (70%) 11 (73.3%)	9 (36%) 12 (30%) 4 (26.7%)	
STONE score: median (IQR)#	6 (6–7)	6 (5.5-7)	0.7

Comparison was done using: *independent samples T test, #Mann-Whitney test, +chi square test, **Fisher’s exact test.

**Table 3:** Analysis of 24-hour urinary metabolic evaluation in relation to patient and stone characteristics

Parameter	Normal 24-hour Urinary Metabolic Evaluation (n = 21)	Abnormal 24-hour Urinary Metabolic Evaluation (n = 59)	*p* value
Gender: N (%)+			0.85
Male Female	14 (26.9%) 7 (25%)	38 (73.1%) 21 (75%)	
BMI (Kg/m^2^): mean ± SD*	18.92 ± 3.99	18.44 ± 3.70	0.62
BMI group: N (%)+			0.12
Normal Obese	14 (22.2%) 7 (41.2%)	49 (77.8%) 10 (58.8%)	
Age at 1st episode (years): median (IQR)#	6 (5–9)	6 (3.5–10)	0.48
Age group: N (%)**			0.88
Toddlers Preschoolers School-aged Teens	4 (28.6%) 6 (30%) 10 (26.3%) 1 (12.5%)	10 (71.4%) 14 (70%) 28 (73.7%) 7 (87.5%)	
Recurrence: N (%)+			0.95
No Yes	13 (26%) 8 (26.7%)	37 (74%) 22 (73.3%)	
Recurrence rate: N (%)**			0.75
None Once More than once	13 (26%) 5 (22.7%) 3 (37.5%)	37 (74%) 17 (77.3%) 5 (62.5%)	
Family history: N (%)+			0.24
Negative Positive	12 (32.4%) 9 (20.9%)	25 (67.6%) 34 (79.1%)	
Urinary tract anomalies: N (%)**			0.75
Absent Present	18 (27.3%) 3 (21.4%)	48 (72.7%) 11 (78.6%)	
Stone volume (cm^3^): median (IQR)#	0.96 (0.36–2.32)	1.49 (0.68–2.79)	0.14
Stone number: median (IQR)#	3 (2–6)	3 (2–4)	0.67
Stone density (HU): mean ± SD*	970.48 ± 406.80	856.53 ± 375.86	0.25
Stone location: N (%)**			0.32
Ureteric Renal Combined Vesical	1 (25%) 12 (21.8%) 7 (36.8%) 1 (50%)	3 (75%) 43 (78.2%) 12 (63.2%) 1 (50%)	
Laterality: N (%)**			0.15
Unilateral Bilateral Vesical	17 (30.9%) 3 (13%) 1 (50%)	38 (69.1%) 20 (87%) 1 (50%)	
Hydronephrosis: N (%)**			0.16
No Grade 1–2 Grade 3–4	8 (32%) 12 (30%) 1 (6.7%)	17 (68%) 28 (70%) 14 (93.3%)	
STONE score: median (IQR)#	6 (6–7)	6 (5.5-7)	0.45

Comparison was done using: *independent samples T test, #Mann-Whitney test, +chi square test, **Fisher’s exact test

**Table 4:** Analysis of combined serum and 24-hour urinary metabolic evaluation in relation to patient and stone characteristics

Parameter	Abnormal Both Serum & Urinary Metabolic Evaluation (n = 19)	Normal Both or Abnormal One of Them (n = 61)	*p* value
Gender: N (%)+			0.85
Male Female	12 (23.1%) 7 (25%)	40 (76.9%) 21 (75%)	
BMI (Kg/m^2^): mean ± SD*	19.70 ± 4.40	18.21 ± 3.49	0.13
BMI group: N (%)**			0.99
Normal Obese	15 (23.8%) 4 (23.5%)	48 (76.2%) 13 (76.5%)	
Age at 1st episode (years): median (IQR)#	6 (4–9)	9 (5.5–9.5)	0.35
Age group: N (%)**			0.58
Toddlers Preschoolers School-aged Teens	10 (71.4%) 18 (90%) 30 (78.9%) 7 (87.5%)	4 (28.6%) 2 (10%) 8 (21.1%) 1 (12.5%)	
Recurrence: N (%)+			0.64
No Yes	11 (22%) 8 (26.7%)	39 (78%) 22 (73.3%)	
Recurrence rate: N (%)**			0.92
None Once More than once	11 (22%) 6 (27.3%) 2 (25%)	39 (78%) 16 (72.7%) 6 (75%)	
Family history: N (%)+			0.91
Negative Positive	9 (24.3%) 10 (23.3%)	28 (75.7%) 33 (76.7%)	
Urinary tract anomalies: N (%)+			0.25
Absent Present	14 (21.2%) 5 (35.7%)	52 (78.8%) 9 (64.3%)	
Stone volume (cm^3^): median (IQR)#	1.62 (0.91–2.79)	1.09 (0.55–2.84)	0.23
Stone number: median (IQR)#	3 (2–5)	3 (2-4.5)	0.6
Stone density (HU): mean ± SD*	916.05 ± 374.14	877.21 ± 390.79	0.7
Stone location: N (%)**			0.62
Ureteric Renal Combined Vesical	1 (25%) 15 (27.3%) 3 (15.8%) 0 (0%)	3 (75%) 40 (72.7%) 16 (84.2%) 2 (100%)	
Laterality: N (%)**			0.99
Unilateral Bilateral Vesical	14 (25.5%) 5 (21.7%) 0 (0%)	41 (74.5%) 18 (78.3%) 2 (100%)	
Hydronephrosis: N (%)**			0.73
No Grade 1–2 Grade 3–4	7 (28%) 8 (20%) 4 (26.7%)	18 (72%) 32 (80%) 11 (73.3%)	
STONE score: median (IQR)#	6 (6–7)	6 (6–7)	0.64

Comparison was done using: *independent samples T test, #Mann-Whitney test, +chi square test, **Fisher’s exact test

### Secondary outcome

Possible risk factors for stone recurrence were assessed. Among the study population, 50 patients (62.5%) had a history of only one stone disease episode. The remaining 30 patients (37.5%) had history of recurrence; 22 (27.5%) were recurrent only once, and 8 (10%) had more than one recurrence episode.

Recurrent and non-recurrent groups were compared regarding the previously assessed patient and stone characteristics (Table 5). In addition, the results of serum and 24-hour urinary metabolic evaluation were compared between both groups (Table 6). There was a significant difference between both groups regarding stone volume, stone number, location, and laterality (*p* = 0.018, 0.002, 0.008, and 0.000, respectively). The recurrence risk was higher with larger stone number and volume. Post-hoc test was done for location and laterality; and the recurrence risk was higher for bilateral (z = 4.8, *p* = 0.000) and combined renal and ureteric (z = 3.2, *p* = 0.001) stones. However, no statistically significant difference was present in relation to serum and urinary metabolic abnormalities.

Cox regression analysis was done to identify significant predictors of recurrence risk in univariate analysis (Table 7). Stone laterality was the only significant predictor of recurrence risk (HR: 3.801, 95% CI: 1.239–11.662, *p* = 0.02) with ~ 3.8 times higher risk of recurrence with bilateral stone disease (78.3% vs. 21.8% with unilateral stone disease).

**Table 5:** Analysis of recurrence risk in relation to patient and stone characteristics.

Parameter	Non-recurrent (n=50)	Recurrent (n=30)	*p* value
Gender: N (%)+			0.47
Male Female	31 (59.6%) 19 (67.9%)	21 (40.4%) 9 (32.1%)	
BMI (Kg/m^2^): mean ± SD*	18.15 ± 3.2	19.26 ± 4.51	0.2
BMI group: N (%)+			0.14
Normal Obese	42 (66.7%) 8 (47.1%)	21 (33.3%) 9 (52.9%)	
Age at 1^st^ episode (years): median (IQR)#	6 (4-10)	5 (3-9)	0.3
Age group: N (%)**			0.25
Toddlers Preschoolers School-aged Teens	7 (50%) 11 (55%) 28 (73.7%) 4 (50%)	7 (50%) 9 (45%) 10 (26.3%) 4 (50%)	
Family history: N (%)+			0.95
Negative Positive	23 (62.2%) 27 (62.8%)	14 (37.8%) 16 (37.2%)	
Urinary tract anomalies: N (%)+			0.65
Absent Present	42 (63.6%) 8 (57.1%)	24 (36.4%) 6 (42.9%)	
Stone volume (cm^3^): median (IQR)#	1.23 (0.57-2.53)	1.7 (0.86-4.23)	**0.018**
Stone number: median (IQR)#	3 (2-5)	4 (3-6)	**0.002**
Stone density (HU): mean ± SD*	855.1 ± 360.46	938.67 ± 423.73	0.35
Stone location: N (%)**			**0.008**
Ureteric Renal Combined Vesical	3 (75%) 39 (70.9%) 6 (31.6%) 2 (100%)	1 (25%) 16 (29.1%) 13 (68.4%) 0 (0%)	
Laterality: N (%)**			**0.000**
Unilateral Bilateral Vesical	43 (78.2%) 5 (21.7%) 2 (100%)	12 (21.8%) 18 (78.3%) 0 (0%)	
Hydronephrosis: N (%)+			0.49
No Grade 1-2 Grade 3-4	18 (72%) 23 (57.5%) 9 (60%)	7 (28%) 17 (42.5%) 6 (40%)	
STONE score: median (IQR)#	6 (5-7)	6 (6-7)	0.97

Comparison was done using: *independent samples T test, #Mann-Whitney test, +chi square test, **Fisher’s exact test.

**Table 6:** Analysis of recurrence risk in relation to serum and 24-hour urinary metabolic abnormalities

Parameter	Non-recurrent (n=50)	Recurrent (n=30)	*p* value
Corrected serum calcium: N (%)**			0.99
Low Normal	4 (57.1%) 46 (63%)	3 (42.9%) 27 (37%)	
Serum phosphorus: N (%)**			0.33
Low Normal High	2 (66.7%) 44 (60.3%) 4 (100%)	1 (33.3%) 29 (39.7%) 0 (0%)	
Serum uric acid: N (%)**			0.36
Normal High	48 (64%) 2 (40%)	27 (36%) 3 (60%)	
Serum pH and HCO_3_: N (%)**			0.57
Low Normal High	6 (60%) 44 (63.8%) 0 (0%)	4 (40%) 25 (36.2%) 1 (100%)	
24-hour urinary calcium: N (%)*			0.81
Normal High	32 (61.5%) 18 (64.3%)	20 (38.5%) 10 (35.7%)	
24-hour urinary uric acid: N (%)**			0.74
Normal High	43 (61.4%) 7 (70%)	27 (38.6%) 3 (30%)	
24-hour urinary oxalate: N (%)*			0.16
Normal High	36 (67.9%) 14 (51.9%)	17 (32.1%) 13 (48.1%)	
24-hour urinary citrate: N (%)**			0.99
Low Normal	4 (66.7%) 46 (62.6%)	2 (33.3%) 28 (37.8%)	
24-hour urine volume: N (%)**			0.29
Low Normal	3 (100%) 47 (61%)	0 (0%) 30 (39%)	
Serum chemistry: N (%)*			0.42
Normal Abnormal	36 (65.5%) 14 (56%)	19 (34.5%) 11 (44%)	
24-hour urine chemistry: N (%)*			0.95
Normal Abnormal	13 (61.9%) 37 (62.7%)	8 (38.1%) 22 (37.3%)	
Combined serum and 24-hour urine chemistry: N (%)*			0.71
Normal Abnormal	10 (66.7%) 40 (61.5%)	5 (33.3%) 25 (38.5%)	

Comparison was done using: *chi square test, **Fisher’s exact test

**Table 7:** Cox regression analysis of recurrence risk predictors

Predictor	Hazard Ratio	95% Confidence Interval		*p* value
		Lower	Upper	
Stone Volume	1.026	0.936	1.125	0.581
Stone number	1.026	0.850	1.239	0.787
Stone location (combined)	1.305	0.532	3.202	0.561
Stone laterality (bilateral)	**3.801**	**1.239**	**11.662**	**0.020**

## Discussion

Pediatric urolithiasis, despite being thought of as an uncommon condition, is becoming increasingly common and significant. According to many recent studies, urolithiasis is becoming more prevalent, especially over the last 25 years [20]. Importantly, almost 50% of pediatric urolithiasis patients recur within 3 years of their first stone episode [21]. All pediatric stone formers are considered a high-risk group among urolithiasis patients due to their high recurrence rate. Following the first stone episode, all children should have a thorough metabolic evaluation performed, according to the most recent American and European guidelines [7, 9].

In this study, we hypothesized that not all children are at high-risk for stone disease recurrence, and routine metabolic evaluation could be refined to include only a high-risk subgroup of pediatric population based on certain patient and stone characteristics. Therefore, we recruited children with history of intervention for calcium-based stone disease. Full metabolic evaluation, including serum and 24-hour urine studies, was performed for included patients.

Regarding the usefulness of serum metabolic assessment in the context of urolithiasis and its relationship to recurrence risk, no consensus has been reached. Serum tests are usually obtained to assess renal function and calcium metabolism. The clinical utility of these tests has been questioned, though, because children with nephrolithiasis rarely have abnormal serum studies [22]. Albeit, serum chemistries that may indicate underlying medical disorders associated with stone disease include creatinine, electrolytes, calcium and uric acid [9].

In a study conducted by Bevill et al. to evaluate metabolic abnormalities in a pediatric population after their first stone episode, a retrospective review of 113 children was performed. Their demographic data, serum and 24-hour urine chemistry results and treatments were compared. When serum chemistry results were evaluated, the likelihood of having serum metabolic abnormalities was very low; and none of the detected abnormalities needed further evaluation or treatment. Hypo- and hypercalcemia were present in 4% and 6% of patients, respectively. Hypo- and hyperphosphatemia were present in 1% and 12%, respectively. Hyperuricemia was present in 4% [22].

In other studies that assessed serum metabolic abnormalities, most patients also had serum chemistry results within normal range. Hypercalcemia, hyperuricemia, and hyperphosphatemia were noted in 2–14%, 3–9% and 2–4%, respectively [23, 24]. To date, no studies have investigated the association between metabolic acidosis and kidney stone formation [25].

In our study, abnormal serum chemistry was present in 25 patients (31.25%). The frequency of relevant metabolic abnormalities was as follows: high serum creatinine (3.75%), hypocalcemia (8.75%) (after correction of serum calcium for hypoalbuminemia), hypo- and hyperphosphatemia (3.75% and 5%, respectively), hyperuricemia (6.25%), metabolic acidosis (12.5%) and metabolic alkalosis (1.25%). These results cope with other studies apart from absence of hypercalcemia in our study population; mostly due to a common, yet non-indicated, practice of dietary calcium restriction.

Regarding 24-hour urinary metabolic abnormalities, hypercalciuria is found in 30–50% of calcium stone formers, making it the most common metabolic abnormality in stone disease patients. Hyperoxaluria is found in 5–30%, hyperuricosuria in 10–40%, and hypocitraturia in 10–50% of patients [26, 27]. Many studies have been conducted to limit the 24-hour urinary metabolic evaluation to assess the metabolites whose abnormalities are of the highest prevalence among stone formers. In the study conducted by Bevill et al., 24-hour urine chemistry results revealed that 99 patients (88%) had at least one abnormal metabolite. This study differs from previous literature findings by identifying a low rate of hypercalciuria and hyperoxaluria (11% and 15%, respectively) and a high rate of low urine volume and hypocitraturia (89% and 68%, respectively), suggesting a change in metabolic abnormalities associated with modern pediatric stone disease [22].

Chan et al. proposed a simplified approach to metabolic evaluation in first-time stone formers with a stone analysis available. A limited urinary metabolic evaluation consisting of four parameters (24-h calcium, citrate, and oxalate and low urinary volume) was compared to a complete urinary metabolic evaluation. These four parameters were selected being the most prevalent metabolic abnormalities reported by previous studies. The number and type of metabolic abnormalities that would have been missed with this limited evaluation were determined. The most common abnormalities were hypocitraturia (69.6%), low urine volume (52.5%), and hypercalciuria (22.5%). Hyperoxaluria and hyperuricosuria were present in 15% and 2.5%, respectively. High pH was present in 33.8% and low pH was present in 15%. A limited urinary metabolic evaluation was then able to detect the most significant metabolic abnormalities. Using this approach, metabolic evaluation can be simplified, and health care costs can be reduced [28].

In a study conducted on an Egyptian population, evaluation of metabolic abnormalities in 24-hour urine revealed metabolic abnormalities in 34 children (23%); with hypercalciuria and hyperoxaluria being the most common (41% and 32%, respectively). Hyperuricosuria and hypocitraturia were present in 21% and 6%, respectively [29].

In our study, abnormal 24-hour urine chemistry was present in 59 patients (73.75%). The frequency of these metabolic abnormalities among our study population was as follows: hypercalciuria (35%), hyperoxaluria (33.75%), low urine pH (31.75%), hyperuricosuria (12.5%), hypocitraturia (7.5%), and low urine volume (3.75%). The main difference from literature was in the prevalence of low urine volume and hypocitraturia; being less in our study population. This difference may be explained by ethnic, dietary, and therapeutic factors.

All the previous trials were on the side of 24-hour urinary metabolic evaluation aiming at finding a simplified or modified approach to overcome its drawbacks. However, another way to get over these drawbacks, we can still utilize 24-hour metabolic evaluation but limit it to a specified group among pediatric stones formers. This group should hence be discriminated from the whole pediatric urolithiasis population by certain risk factors. So, in this study, the results of serum and 24-hour urinary metabolic evaluation were tested for association with relevant patient and stone characteristics and were analyzed in relation to them. This approach aiming at modifying metabolic evaluation for pediatric urolithiasis wasn’t utilized in any previous studies. Unfortunately, the results of our analysis failed to identify such a high-risk group. None of the assessed patient and stone characteristics revealed a significant association with abnormal groups of serum, 24-hour urine or combined serum and 24-hour urine metabolic evaluation.

We also assessed recurrence risk for possible association with patient and stone characteristics, and with serum and urinary metabolic abnormalities. To date, most studies describe factors contributing to de novo urolithiasis; however, factors related to recurrence risk were also assessed in a few publications. In a retrospective study by De Ruysscher et al. to identify risk factors associated with recurrent nephrolithiasis in pediatric population, patient characteristics, presenting symptoms, medical history, laboratory results and management strategy (conservative vs. surgical) were analyzed. Only immobilization and need for surgical intervention were associated with a higher risk of stone recurrence in univariate, but not in multivariate analysis [30].

In another study by DeFoor et al., there was a significant difference in urinary calcium and citrate levels between children with de novo and recurrent calcium-based stone disease [31]. However, in a multi-center, multi-model, externally validated machine-learning study, routine urinary metabolic evaluation didn’t accurately predict stone type nor recurrence in nephrolithiasis patients [32].

Our results cope with those of De Ruysscher et al. and machine-learning study regarding the association between metabolic abnormalities and recurrence risk. However, immobilization, as a risk factor, was not assessed in our study. Management strategy (conservative vs. surgical) also couldn’t be assessed as our study included only patients who had surgical intervention. Stone characteristics were not previously explored as a risk factor for stone disease recurrence, to our knowledge. In our study, some stone characteristics (including stone volume, number, location, and laterality) had a significant association with the risk of recurrence in univariate analysis. However, in multivariate analysis, only stone laterality revealed such a significant association. Identifying a subgroup with high recurrence risk will help to provide them with more intense follow-up after the first stone episode.

After all, our findings were based on a small study population with a non-prospective, non-controlled design. Also, repeating 24-hour urine collection is recommended by guidelines to reflect metabolic variability and overcome dietary effects; however, in view of poor patient recruitment and compliance, and due to limited resources, 24-hour urinary metabolic evaluation was performed only once. So, we recommend that relevant patient and stone characteristics could be further assessed at a larger scale, prospective, controlled trials hoping to find a significant association between any of them and serum and urinary metabolic abnormalities. Furthermore, regarding recurrence risk, the follow-up period for some children might not have been long enough to observe all possible recurrences.

## Conclusion

Routine serum and 24-hour urinary metabolic evaluation is still indicated in all pediatric calcium-based stone formers; being a high-risk population. However, the frequency of 24-hour urinary metabolic abnormalities is much higher than those in serum (73.75% and 31.25%, respectively). Furthermore, the recurrence risk is higher in patients with bilateral urolithiasis. So, in case of limited resources, metabolic evaluation could be restricted to 24-hour urine only and to patients with bilateral urolithiasis.

## Data Availability

No datasets were generated or analysed during the current study.
